# Negative axial strain sensitivity in gold-coated eccentric fiber Bragg gratings

**DOI:** 10.1038/srep38042

**Published:** 2016-11-30

**Authors:** Karima Chah, Damien Kinet, Christophe Caucheteur

**Affiliations:** 1 Electromagnetism and Telecommunication Department, University of Mons (UMONS), Boulevard Dolez, 31, 7000 Mons, Belgium

## Abstract

New dual temperature and strain sensor has been designed using eccentric second-order fiber Bragg gratings produced in standard single-mode optical fiber by point-by-point direct writing technique with tight focusing of 800 nm femtosecond laser pulses. With thin gold coating at the grating location, we experimentally show that such gratings exhibit a transmitted amplitude spectrum composed by the Bragg and cladding modes resonances that extend in a wide spectral range exceeding one octave. An overlapping of the first order and second order spectrum is then observed. High-order cladding modes belonging to the first order Bragg resonance coupling are close to the second order Bragg resonance, they show a negative axial strain sensitivity (−0.55 pm/με) compared to the Bragg resonance (1.20 pm/με) and the same temperature sensitivity (10.6 pm/°C). With this well conditioned system, temperature and strain can be determined independently with high sensitivity, in a wavelength range limited to a few nanometers.

The use of femtosecond laser pulses has emerged as a competitive technology for Fiber Bragg Gratings (FBGs) inscription. The main advantage of this kind of lasers is the capability to engrave directly a variety of structures in almost all types of optical materials. Indeed, it has been demonstrated that femtosecond laser beam can be focused below the diffraction limit such that structures as small as 240 nm can be obtained^1^,^2^. With femtosecond pulses, the refractive index modulation is induced via a multi-photon transition process^3^. The latter does not require the presence of doping in the material such as germanium in silica matrix involved in traditional inscription methods based on continuous and nanosecond UV lasers. Thereby, the use of long working distance high numerical aperture microscope objective and subnanometric precision translation stages allow controlling the position of the inscribed grating in the core of the optical fiber^4^,^5^,^6^,^7^. Birefringent gratings can then be produced when the induced refractive index modulation is slightly shifted from the fiber core center, creating an asymmetry in the fiber cross section^4^,^8^,^9^. In these FBGs, also called eccentric gratings, both the Bragg and cladding modes are excited^4^,^10^. This is manifested by a transmission amplitude spectrum with the Bragg resonance at the right-hand side and several hundreds of narrowband dips (bandwidth ~200 pm) corresponding to the cladding modes at its left side. Their spectral range was estimated to one octave^10^, which is usually much more than tilted fiber Bragg gratings (TFBGs). Furthermore, these gratings can operate at very high temperature (more than 1000 °C) without any degradation of the FBGs performances, as it is the case for type II or type IIA FBGs^11^,^12^.

Beyond their use for lasers and telecommunication applications, the integration of FBGs as sensors has become almost a routine nowadays. Nevertheless, gratings that can be used in harsh environment with reliable self-compensation sensing solutions are still relevant to produce, especially for structural health monitoring, nuclear and oil & gas industries^13^,^14^. As FBGs in standard optical fibers are sensitive to both strain and temperature, different techniques have been set-up to discriminate between these two measurands. To cite but a few: FBGs in the same optical fiber where one is protected from thermal effect^15^, two gratings inscribed at two different wavelengths achieving different sensitivities^16^, a single FBG measured at its first and second order Bragg wavelength^17^, Bragg mode and cladding modes of standard tilted FBGs^18^, FBG in temperature insensitive specialty fibers^19^, among others^13^,^20^.

In this work, we demonstrate the production of first- and second-order eccentric FBGs with the 800 nm infrared (IR) femtosecond point-by-point (PbP) inscription technique. Our purpose is to study the sensing properties of the Bragg and cladding modes resonances in a wide spectral range (300 nm). These cladding modes are highly influenced by the surrounding medium. Hence, below the cut-off wavelength (corresponding to the resonance for which the effective refractive index matches the one of the surrounding medium), cladding modes are radiated^21^. They therefore disappear from the amplitude spectrum. In air, this phenomenon appears about 250 nm below the Bragg wavelength. In the following, we show that a thin gold coating deposited on the fiber surface at the grating location can prevent this radiation. Moreover, we demonstrate the existence of preserved cladding mode resonances over the predicted spectral range of one octave. Indeed, in the case of gold-coated second-order gratings, high-order resonances of the first-order grating overlap with the ones of the second-order grating. We experimentally demonstrate that while axial strain sensitivity of these cladding modes is mode-order dependent, temperature sensitivity is wavelength dependent, i.e. modes at the same wavelength range exhibit the same temperature sensitivity whatever they originate from first or second-order core mode coupling. However, they have totally different strain sensitivities.

As a result of these couplings, it is possible to monitor (in a short spectral range of several nanometers) both second order Bragg peak and high order cladding modes. While both kinds of peaks exhibit a similar temperature sensitivity (~10 pm/°C in the considered spectral window), their axial strain sensitivity is completely different (1.2 pm/με and −0.55 pm/με, respectively). These features yield one of the best-conditioned systems reported so far. Other important practical benefits result from the flexibility of production and the inherent grating resistance to high-temperature environments.

## Experiments

**Figure 1:**
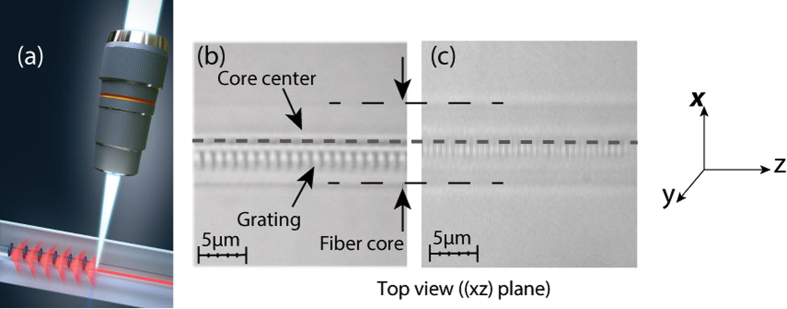
(**a**) Principle of PbP inscription technique: 800 nm Femtosecond laser beam is focused with a microscope objective into the optical fiber core. (**b**) Transmission microscope images of 2 μm eccentricity second order FBG compared to 1 μm eccentricity first order FBG (**c**).

The production of eccentric FBGs is carried out with the direct PbP inscription technique and 800 nm femtosecond pulses delivered by Spitfire pro Spectra-Physics amplifier of 120 fs duration and 1 kHz repetition rate. The experimental setup and inscription process were described in previous works^4^,^9^. It consists in focusing the laser beam in the fiber core with high numerical aperture (0.65) microscope objective and without oil immersion (Fig. 1a). The fiber is then displaced along its z optical axis at a constant speed using a high precision air bearing translation stage to create a uniform modification over the required length. Each focused femtosecond pulse induces a refractive index modification in a point-like region of the fiber core, as shown in Fig. 1b.

The spacing between these points corresponds to the grating period, which can be controlled by correlating the speed of the translation stage to the repetition rate of the laser. To produce eccentric gratings, the fiber was first shifted 2 μm off-center in the horizontal direction (x direction as shown in Fig. 1), which was visually controlled in reflection using an infrared camera positioned above the microscope objective. The fiber was then translated with a constant speed along the fiber z axis up to a length of 1 cm. Figure 1b shows the transmission microscope image of 2 μm eccentricity second order FBG compared to 1 μm eccentricity first order FBG obtained by a high resolution (<300 nm) optical microscope (Zeiss Axio Imager.M2m). We have inscribed complementary gratings from the point of view of cladding modes orders beyond the cut-off wavelength (λ_C_) in the spectral range [1300 nm–1600 nm] covered by the broadband optical source (Amonics Superluminescent Diode ASLD-CWDM-5-B-FA) and spectrum analyser (Yokogawa AQ6370) used in this work. In the following, we report the behaviour of 3 gratings inscribed in standard single mode optical fibre (Corning SMF28) with different periods 550 nm, 633 nm and 1096 nm and the same eccentricity (2 μm). Table 1 summarizes their main features, in terms of Bragg and cut-off wavelengths.

**Figure 2:**
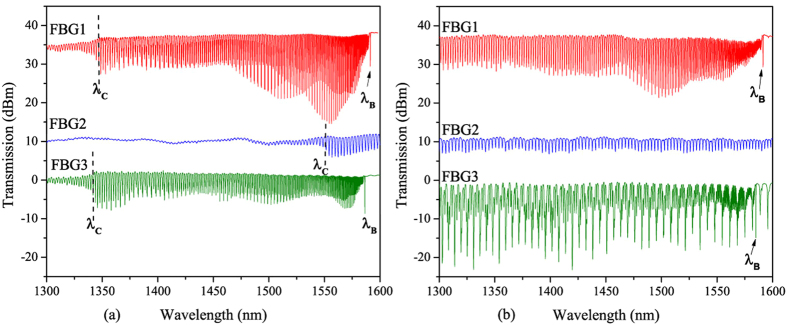
Transmission spectrum of eccentric gratings FBG1, FBG2 and FBG3 with 550 nm, 633 nm and 1096 nm periods respectively measured (**a**) in air and (**b**) with a 50 nm gold coating on the cladding surface. Bragg wavelength (λ_B_) and cut-off wavelength (λ_C_) are indicated.

Figure 2a shows their transmitted amplitude spectra in air. For FBG1, we can see the first order Bragg resonance (λ_B_) occurring at 1591 nm, and at its left side, in a very huge spectral range, cladding modes resonances, which are drastically attenuated around λ_C_ = 1345 nm (cut-off wavelength) indicted by a dashed line. In FBG2, we can only see cladding modes resonances down to λ_C_ = 1548 nm, because the Bragg resonance is out of the spectral range (1831 nm). FBG3 shows a similar transmitted amplitude spectrum as FBG1, with 1585 nm second order Bragg resonance and 1341 nm cut-off wavelength.

It is worth mentioning that the spectra are composed by four types of cladding modes TE0m, TM0m, HElm, and EHlm, where “l” is the azimuthal order and “m” is the order of a mode. The radial (Er) and azimuthal (Eϕ) electric fields are null for TE0m and TM0m modes so that they are polarization-independent. However, both HElm and EHlm modes are polarization-dependent since they combine axial and transverse components. As a result, a x-polarized core mode couples to the even HElm and TE0m modes while a y-polarized one couples to the odd EHlm and TM0m modes. Highest order modes EHlm and HElm are dominantly radially and azimuthally polarized, respectively^22^.

Each *j*^th^ cladding mode resonance resulting from core mode coupling to cladding appears at a wavelength 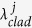 given by:


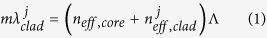


Where m is the grating order, *n*_*eff*,*core*_ and 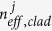 are, respectively, the effective refractive indices of the core and the *j*^th^ cladding mode and 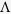 is the period of the grating.

The cut-off wavelength can be easily calculated from Eq. (1). For silica fiber *n*_*eff*,*core*_ = 1.4467, the cut-off wavelength in air occurs around 1345 nm for FBG1, at 1545 nm for FBG2 and at 1340.7 nm for the second order grating FBG3. These values are in good agreement with the reported experimental results (Fig. 2). We must recall that within the framework of this study, sensing properties of the Bragg peak and cladding modes resonances have to be determined versus cladding modes order or more precisely the corresponding effective refractive index deduced from Eq. (1). In the considered spectral range (1300 nm, 1600 nm), if the cladding modes amplitudes are all maintained, FBG1 should give access to the core mode and cladding modes with *n*_*eff*,*clad*_ down to 0.9165. For FBG2 we can have cladding modes with *n*_*eff*,*clad*_ from 1.0732 down to 0.6007, and second order FBG3 will give rise to second order cladding modes with the same *n*_*eff*,*clad*_ as in FBG1. Therefore, so as to preserve cladding modes amplitudes below the cut-off wavelength, FBGs were shielded from the outer medium by a thin gold sheet (~50 nm) deposited on the fiber surface. A sputter coater was used for this purpose. The coated gratings were then cured at 200 °C during 6 hours for improved gold adhesion^23^.

Figure 2b shows the transmitted amplitude spectrum of these gold-coated eccentric gratings. Compared to Fig. 2a, we can clearly see that the gold sheet protection allows to recover cladding modes below the cut-off wavelength for the two first FBGs while for the third grating, two sets of modes become superimposed, the ones already present in Fig. 2 and others more spaced between each other.

**Figure 3:**
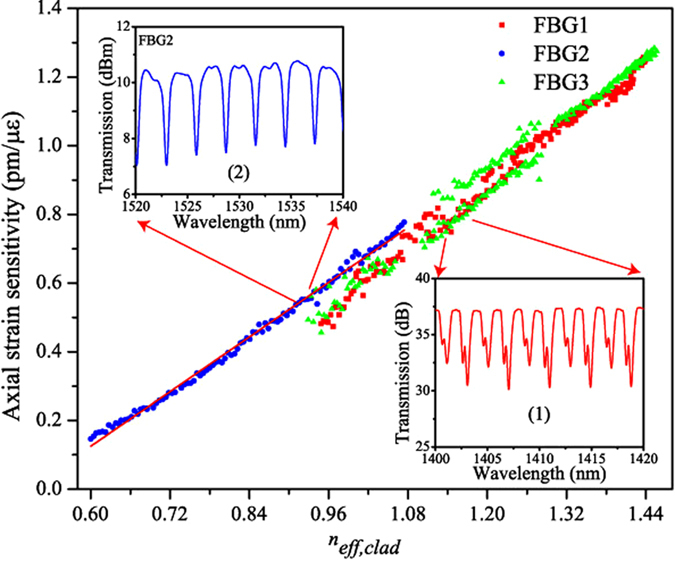
Axial strain sensitivity versus the effective index of the cladding modes for excentric gratings FBG1, FBG2 and FBG3. Inset (1) and Inset (2) transmitted amplitude spectra for FBG1 and FBG2 respectively.

According to other studies^7^ these modes can be identified as high order modes from the first order Bragg resonance coupling. From FBG1 and FBG2 spectra (Fig. 3), it appears that when the cladding modes are shielded from the outer medium influence, they can extend in very wide spectral range and even overlap on the second order spectrum. The advantage of having these high order cladding modes spectrally close to the Bragg resonance can be exploited to study simultaneously their sensitivities to axial strain and temperature.

It has been previously demonstrated that in tilted^21^,^24^,^25^ and highly tilted (80 degree) FBGs^26^, high order modes show differential strain sensitivity. Nevertheless, in these gratings Bragg resonance and high order modes that achieve the highest differential sensitivity are spectrally far (more than 100 nm). In this work, we have the opportunity with these special gratings to study the axial strain sensitivity for more extended mode-orders (FBG1 and FBG2) and even simultaneously with the Bragg reflection in FBG3.

**Figure 4:**
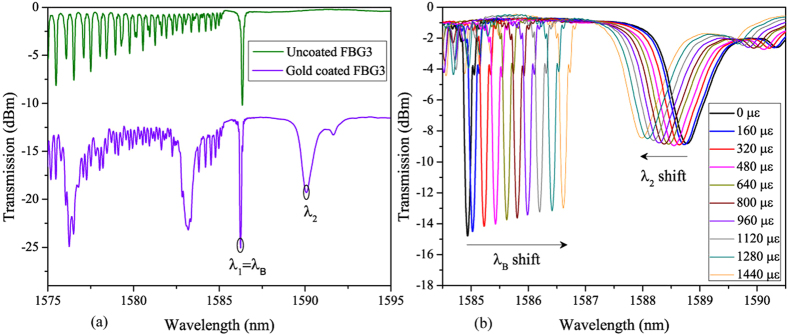
(**a**) Transmission spectrum of second-order grating FBG3 around the Bragg resonance (**a**) comparison between gold-coated and uncoated grating (**b**) Influence of axial strain.

Figure 3 illustrates the axial strain sensitivity versus the effective refractive index of the cladding modes deduced from Eq. (1) for the 3 investigated gratings. Eccentric gratings break the cylindrical symmetry of the fiber cross-section and locally creates a strong form birefringence^4^,^10^. For all the measurements, we have used a broadband source (Superluminescent Diodes Amonics ASLD-CWDM-5-B-FA) and spectrum analyser (Yokogawa AQ6370). A polarization controller was used to maintain the polarization of the light parallel to the fast mode of the grating. The plot shows a linear trend for the axial strain sensitivity versus *n*_*eff*,*clad*_. We can see clearly an interval of *n*_*eff*,*clad*_ between 0.95 and 1.28 where the dispersion in the measurements increases for FBG1 and FBG3. In fact this region partly shown in the inset of Fig. 3, corresponds to high-order modes that display doublets. Indeed different families of cladding modes with different polarisation dependence compose the transmission amplitude spectrum of eccentric FBGs^10^. These families of modes may undergo different sensitivities as it was demonstrated in standard tilted FBGs for radially and azimuthally polarized modes^24^. Therefore, we assume that the peaks composing the doublets belong to two different families of modes with slightly different axial strain sensitivities. In the case of FBG2, the peaks are well defined (cf. upper inset of Fig. 4), yielding a linear dependence of the axial strain sensitivity versus *n*_*eff*,*clad*_.

The linear regression of the experimental data for FBG2 yields:


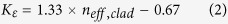


where *K*_*ε*_ is the strain sensitivity expressed in pm/με.

According to Eq. (1) and (2), one can assume that cladding modes with very low effective refractive index can achieve a negative axial strain sensitivity. Unfortunately, Eq. (1) is not sufficient to evaluate the effective refractive index overlapping on the second order Bragg spectrum. Numerical simulations conducted with the FimmWave complex mode solver for the optical fiber configuration used in this work confirm that hundreds of cladding modes can be supported with effective refractive indices ranging between 1.44 and 0.10. The spacing between neighbouring modes increases with the mode order. Considering that *n*_*eff*,*clad*_ = 0.1 in Eq. 2 yields a negative strain sensitivity of −0.66 pm/με. Such negative values for strain sensitivity have been already reported for high-order cladding modes in highly tilted (80 degree) FBGs^26^ and long period gratings^27^.

Therefore, gold-coated FBG3 is a perfect illustration of the presence of very high cladding modes (with very low *n*_*eff*,*clad*_) coexisting with the Bragg resonance. In the following, we will study axial train and temperature sensitivities of this eccentric second-order grating.

## Axial strain sensing properties

To decouple between temperature and strain effects, we propose to use here the second order Bragg resonance (Peak 1 at λ_1_) and the nearest high order cladding mode resonance (Peak2 at λ_2_) in the gold-coated eccentric second order FBG3. Figure 4a displays a close-up around the Bragg resonance of uncoated FBG3 transmission amplitude spectrum in air compared to the gold-coated one. It shows the selected two modes for temperature and strain characterization. The demodulation technique is based on the well-known matrix inversion approach^28^.

When axial strain and temperature are modified in an optical fiber, the wavelength shift is expressed as:


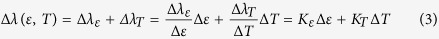


where Δλ_ε_ and Δλ_T_ are the wavelength changes under strain and temperature and *K*_*ε*_, *K*_*T*_ are the strain and the temperature sensitivity coefficients, respectively.

Temperature and strain can be determined independently if their corresponding sensitivity coefficients of the considered peaks (1 and 2) satisfy the good conditioning of the matrix inversion:


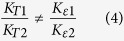


**Figure 5:**
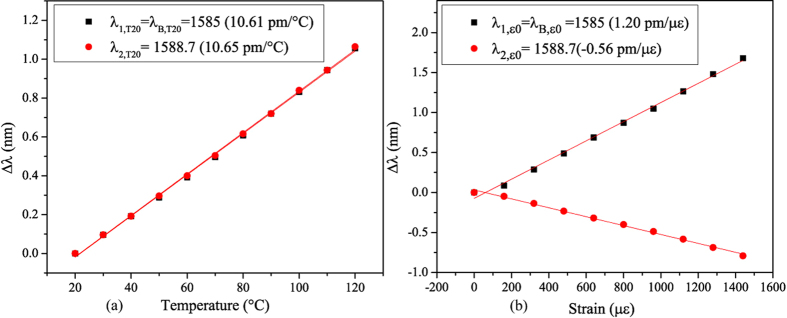
Axial strain (**a**) and temperature (**b**) dependence for the Bragg mode and one high order cladding mode (peak 2 Cf. Fig. 4).

The calibration curves for temperature and strain measurements of FBG3 allow extracting the sensitivities coefficients. In Fig. 4b we report the transmission amplitude spectrum of FBG3 around the Bragg resonance for different applied axial strains. It shows clearly that the Bragg peak at λ_B_ and cladding mode peak at λ_2_ undergo opposite shifts when the axial strain increases. The linear regression of the experimental data depicted in Fig. 5a gives a sensitivity of *K*_*ε*1_ = 1.20 pm/με for the Bragg peak whereas the one for high order cladding mode is *K*_*ε*2_ = −0.56 pm/με. This negative value is highly different from the Bragg peak sensitivity, which completely fulfill the condition of Eq. (4).

For temperature calibration, the slope of the linear regression for the experimental wavelength shift versus temperature data (Fig. 5b) gives almost the same value (10.06 pm/°C and 10.05 pm/°C for *K*_*T*1_ and *K*_*T*2_, respectively).

For sensing application, a performance factor to compare the efficiency of decoupling methods is proposed in ref. 29. Accordingly, the experimentally determined sensitivities coefficients lead to a decoupling factor of 94%. This very high value places the proposed sensor among the most effective ones.

## Conclusion

Eccentric FBGs were produced by point-by-point direct writing technique with femtosecond pulses. The flexibility and tunability of this inscription technique provide gratings with different pitches, which gives the possibility to study a very wide spectral range transmission amplitude spectrum providing access to different cladding mode orders. We experimentally demonstrate that the axial strain sensitivity of the cladding modes decreases linearly with the effective refractive index of the mode. For very high cladding mode orders that are kept in the amplitude spectrum thanks to a gold overlay, negative values can even be reached. This high differential strain sensitivity was exploited to implement a sensor for independent temperature and strain measurements from a single eccentric second-order grating. This sensor considers the sensing properties of the Bragg grating core mode and one very high-order cladding mode. We show that this cladding mode exhibits a similar temperature sensitivity (10.6 pm/°C) but a strongly different strain (−0.55 pm/με) sensitivity compared to the Bragg peak (10 pm/°C and 1.20 pm/με respectively). These properties are considered ideal for dual sensing purposes.

## Additional Information

**How to cite this article**: Chah, K. *et al*. Negative axial strain sensitivity in gold-coated eccentric fiber Bragg gratings. *Sci. Rep.* **6**, 38042; doi: 10.1038/srep38042 (2016).

**Publisher's note:** Springer Nature remains neutral with regard to jurisdictional claims in published maps and institutional affiliations.

## Figures and Tables

**Table 1:** Physical parameters of eccentric FBGs produced by IR femtosecond PbP technique.

Grating	Period	Order: m	Bragg resonance	Cut-off wavelength
FBG1	550 nm	1	1591 nm	1345.9 nm
FBG2	633 nm	1	1831 nm	1548.6 nm
FBG3	1096 nm	1	3168 nm	2680 nm
		2	1585 nm	1341.0 nm
